# Nutritional status and prevalence of anemia in patients with Crohn’s disease after surgery: a multicenter cross-sectional study in China

**DOI:** 10.3389/fnut.2025.1674853

**Published:** 2025-10-15

**Authors:** Yu Shen, Huaying Liu, Jing Liu, Changling Tang, Zhijian Liu, Xujie Dai, Wei Liu, Qian Cao, Xiaolong Ge, Wei Zhou

**Affiliations:** ^1^Department of Nutrition and Food Safety, Zhejiang Provincial Center for Disease Control and Prevention, Hangzhou, China; ^2^Department of Medicine, Guangxi Health Science College, Guangxi, China; ^3^Department of Gastroenterology, School of Medicine, Sir Run Run Shaw Hospital, Zhejiang University, Hangzhou, Zhejiang, China; ^4^Department of Colorectal Surgery, Deqing People’s Hospital, Huzhou University, Zhejiang, China; ^5^Department of General Surgery, Nanjing Drum Tower Hospital, The Affiliated Hospital of Nanjing University Medical School, Nanjing, Jiangsu, China; ^6^Department of General Surgery, Nanjing Jinling Hospital, Affiliated Hospital of Medical School, Nanjing University, Nanjing, Jiangsu, China; ^7^Department of General Surgery, School of Medicine, Sir Run Run Shaw Hospital, Zhejiang University, Hangzhou, Zhejiang, China

**Keywords:** Crohn’s disease, surgery, malnutrition, anemia, a multicenter cross-sectional study

## Abstract

**Background:**

Malnutrition and anemia are major concerns that significantly impact quality of life and disease activity in patients with Crohn’s disease (CD) following surgical resection. However, comprehensive data on nutritional status and anemia in post-surgical CD patients remain limited. This study aims to evaluate the prevalence of malnutrition and anemia in a multi-center cohort of CD patients after surgery.

**Methods:**

In this cross-sectional study, patients with CD who underwent bowel resection across 20 provinces in China were evaluated for nutritional status and anemia. Biochemical parameters were collected and analyzed to explore their associations with nutritional status and anemia.

**Results:**

A total of 160 patients, with a mean age of 37.6 years and 63.8% male, were enrolled. Malnutrition was observed in 47.5% of patients, with 56.3% at risk of malnutrition post-surgery. Malnourished patients exhibited lower nutritional indicators and more severe disease activity. Anemia was detected in 60.6% of patients, with 79.4% having mild anemia and 20.6% moderate anemia. Patients with post-surgical anemia showed elevated inflammatory markers and increased disease activity. Both malnutrition and anemia were significantly associated with reduced quality of life (*p* < 0.05).

**Conclusion:**

Malnutrition and anemia were highly prevalent and negatively impacted patients with CD following surgery. Screening and early preventive management of malnutrition and anemia were critical components of postoperative care in CD.

## Introduction

The incidence of inflammatory bowel disease (IBD) has risen steadily not only in Western countries but also in Asia (1). IBD encompasses Crohn’s disease (CD) and ulcerative colitis (UC), both of which are chronic inflammatory disorders of the gastrointestinal tract. Recent studies have implicated genetic factors, environmental triggers, dietary influences, and alterations in the gut microbiota as key contributors to IBD flares (2). To date, however, the precise pathogenesis of IBD remains elusive. In recent years, treatments for CD drawing from both basic and clinical research have undergone dramatic evolution. Nevertheless, approximately 70% of CD patients will ultimately require surgical resection owing to penetrating or stenosis complications over in course of their lifetime (3).

Nutritional status in CD patients is closely associated with disease activity, and undernutrition is a common complication (4). The etiology of malnutrition in CD is multifactorial, encompassing intricate pathophysiological mechanisms. The predominant factor is diminished oral intake secondary to heightened disease activity (5). Other contributors include postprandial discomfort, diarrhea, prior bowel resections, accelerated gastrointestinal transit, malabsorption, systemic inflammation, and metabolic perturbations induced by pharmacotherapy (6).

Anemia represents another prevalent extraintestinal manifestation of CD, contributing to diminished quality of life and heightened mortality risk (7). Numerous investigations have assessed anemia prevalence in IBD, revealing rates of 9–73% among outpatients and 32–74% among hospitalized patients (8). The elevated anemia burden in IBD is attributed to iron deficiency, protracted chronic inflammation, adverse medication effects, diarrhea, Vitamin B_12_ deficiency, and malabsorption (9). Anemia prevalence is greater in CD than UC (8). In light of anemia’s high incidence and its linkage to disease activity, the European Crohn’s and Colitis Organisation (ECCO) has endorsed routine anemia screening for IBD patients since 2007 (10).

However, to the best of our knowledge, lack of multicenter data on nutrition and anemia in post-surgical CD patients in China. The aim of this study was to evaluate not only the prevalence of malnutrition but also the prevalence of anemia among multi-center cohorts of postoperative CD patients. The findings of this study will inform strategies to sustain remission in CD and mitigate the risk of surgical recurrence.

## Materials and methods

### Study design

This was a cross-sectional baseline study investigating the nutritional status and prevalence of anemia in CD patients with a history of surgical resections for 6 months to 12 months post-surgery. The study was conducted between June 2023 and December 2023, involving CD patients from 20 provinces in China (Zhejiang, Jiangsu, Hubei, Heilongjiang, Fujian, Guangdong, Guangxi, Jiangxi, Anhui, Beijing, Liaoning, Shanxi, Chongqing, Tianjin, Yunnan, Jilin, Hunan, Shanghai, Henan, Hebei). A standardized and uniform protocol was followed across all selected hospitals in these provinces.

**Figure 1:**
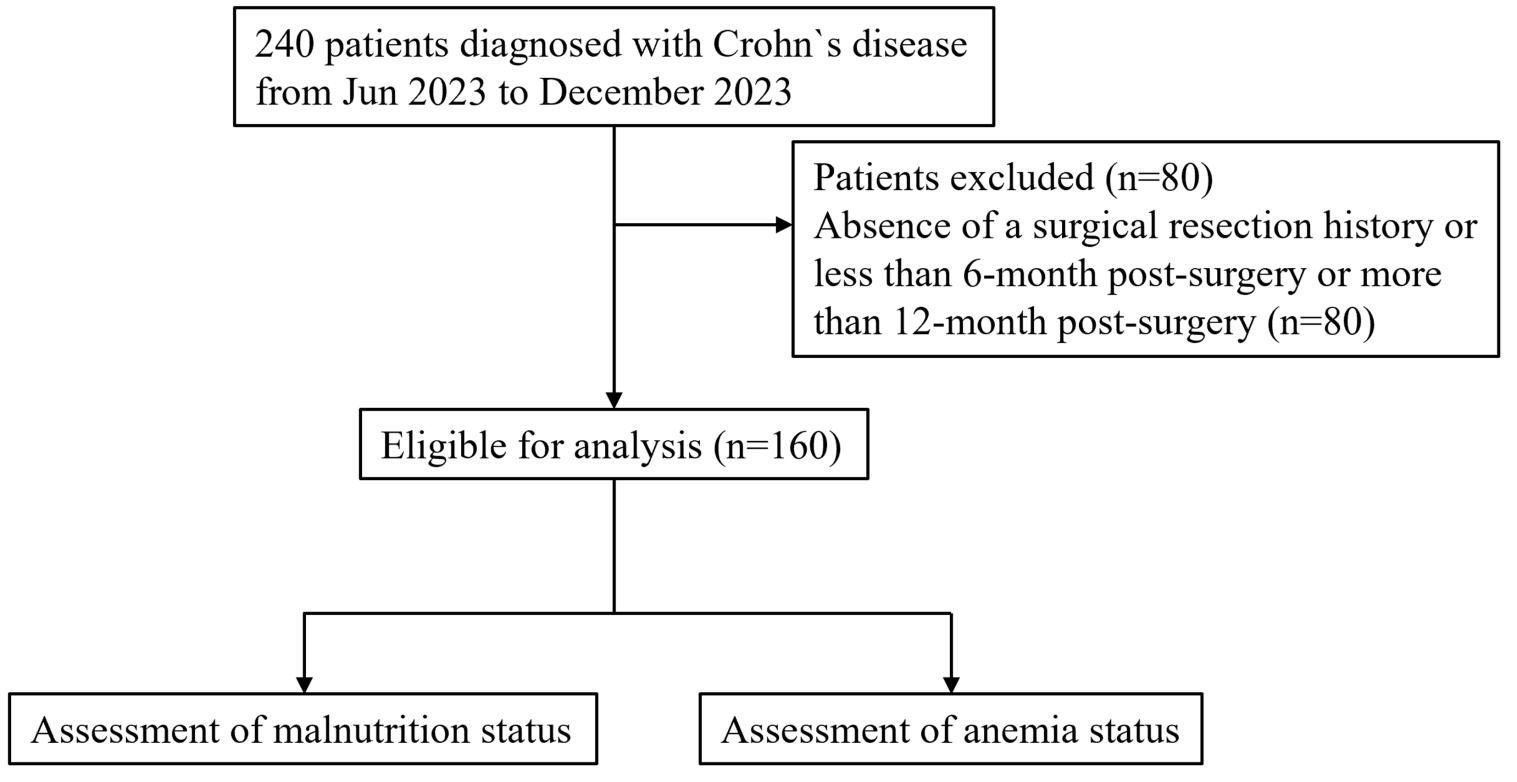
A flow diagram for patients recruitment and exclusion.

The study included patients with a radiologic, endoscopic, and histological diagnosis of CD, based on ECCO guidelines, who had undergone bowel resection due to complications or failure of medical therapy (11). A total of 160 CD patients with a history of surgery were recruited to evaluate their nutritional status and the prevalence of anemia post-surgery. This study was in accordance with the principles of the Declaration of Helsinki and was approved by the Ethics Committee of each participating hospital. Written informed consents were obtained from all patients enrolled in this study. A flow diagram was shown in Figure 1.

### Data collection

Data were collected using a standardized questionnaire developed by the IBD center and the Department of Nutrition. The questionnaire consisted of three sections. The first section captured baseline characteristics of CD patients, including age, sex, smoking habits, marital status, Montreal classification, disease duration, disease activity (assessed using the Crohn’s Disease Activity Index, CDAI), the Inflammatory Bowel Disease Questionnaire (IBDQ) for quality of life assessment, past medications, and current therapies. The second section focused on laboratory data collected within 2 weeks of the interview, including C-reactive protein (CRP), erythrocyte sedimentation rate (ESR), red blood cell (RBC) count, hemoglobin (Hb), hematocrit (Hct), white blood cell (WBC) count, neutrophil and lymphocyte counts, calcium (Ca), platelet (PLT) count, albumin (ALB), and pre-albumin (PAB). The third section addressed nutritional status, covering weight, height, body mass index (BMI), Nutritional Risk Screening score (NRS-2002), fat-free mass index (FFMI), and the Subjective Global Assessment (SGA) score (12, 13). The SGA tool, used for nutritional assessment based on medical history and physical examination, categorized patients into three grades: A (well-nourished), B (moderately malnourished), and C (severely malnourished) (6).

### Definitions of outcomes

#### Malnutrition

Malnutrition was defined as meeting any of the following criteria (6): (1) BMI<18.5 kg/m^2^; (2) FFMI lower than the 10th percentile; (3) SGA grade B or C.

#### Assessment of the nutritional risk

NRS-2002 was assessed for the nutritional screening of CD patients according to ESPEN guidelines (12). The NRS-2002 score was calculated based on impaired nutritional status, disease severity, and age (14). Patients were considered at high nutritional risk if they had an NRS-2002 score ≥ 3.

#### Body mass index

According to the World Health Organization (WHO)’s definition of obesity, BMI were classified into four parts: underweight: <18.5 kg/m^2^; normal: 18.5–25 kg/m^2^; overweight: 25–29.9 kg/m^2^; obesity: >30 kg/m^2^.

#### Anemia

Anemia was defined by the WHO based on age and sex, with hemoglobin levels less than 120 g/L in nonpregnant women and less than 130 g/L in men (7).

#### Ethics statement

This study was approved by the ethics committee of each participating hospital, and written informed consents were obtained from all patients enrolled in this study.

#### Statistical analysis

All of the statistical analyses were performed using SPSS 21.0 (SPSS, Inc., Chicago, IL). Continuous data was presented as the mean ± SE or median (range), while categorical data were presented as number (%). Continuous variables were analyzed using the student *t* test or Mann–Whitney U test depending on the normality of the data distribution, and the categorical variables were analyzed using Pearson χ^2^ test or Fisher exact test, as appropriate. *p* value <0.05 was considered to be statistically significant.

## Results

A total of 240 Crohn’s disease (CD) patients from 43 hospitals across 20 provinces participated in this multicenter cross-sectional study. Eighty patients were excluded due to the absence of a surgical resection history attributable to CD complications or failure of medical therapy, leaving 160 CD patients for analysis.

### Basic characteristics of CD patients

Among the 160 CD patients, 102 (63.8%) were male and the mean age was 37.6 years. Thirty-one patients (19.4%) were smokers, and 122 (76.3%) were married. All patients had undergone surgical resection, with 2.5% sigmoidectomy, 16.9% right hemicolectomy, 1.9% left hemicolectomy, 4.4% a total or segmental colectomy, 38.1% ileocolectomy, and 36.3% small bowel resection. At the time of assessment, most patients were receiving thiopurine or infliximab therapy to prevent postoperative recurrence. Additionally, nearly half had undergone nutritional therapy following surgery, including oral nutritional supplements (ONS) and enteral nutrition (EN) delivered via nasogastric tube. Micronutrient and vitamin supplementation was administered to 24.4 and 33.1% of patients, respectively. Further details are presented in Table 1.

**Table 1:** Baseline characteristics of the patients with and without malnourished.

Variable	Total (160)	Normal (84)	Malnourished (76)	*p*-value
Male, *n* (%)	102 (63.8)	56 (66.7)	46 (60.5)	0.362
Mean age, years	37.6 ± 1.0	38.0 ± 1.5	37.1 ± 1.3	0.656
Mean duration of CD, month	80.6 ± 5.1	78.4 ± 7.4	83.1 ± 6.9	0.648
Smokers, *n* (%)	31 (19.4)	16 (19.1)	15 (19.7)	0.945
Age (≤16), *n* (%)	2 (1.3)	0	2 (2.6)	0.227
Age (17–40), *n* (%)	99 (61.9)	57 (67.9)	42 (55.3)	0.083
Age (>40), *n* (%)	59 (36.9)	27 (32.1)	32 (42.1)	0.216
L1 (ileal), *n* (%)	46 (28.8)	25 (29.8)	21 (27.6)	0.727
L2 (colonic), *n* (%)	18 (11.3)	15 (17.9)	3 (3.9)	0.005
L3 (ilecolonic), *n* (%)	90 (56.3)	40 (47.6)	50 (65.8)	0.027
L4 (upper gastrointestinal), *n* (%)	15 (9.4)	9 (10.7)	6 (7.9)	0.524
B1 (failure of medical therapy), *n* (%)	35 (21.9)	20 (23.8)	15 (19.7)	0.506
B2 (structuring), *n* (%)	59 (36.9)	31 (36.9)	28 (36.8)	0.943
B3 (penetrating), *n* (%)	53 (33.1)	25 (29.8)	28 (36.8)	0.373
B2 + B3, *n* (%)	13 (8.1)	8 (9.5)	5 (6.6)	0.481
Perianal disease, *n* (%)	44 (27.5)	26 (31.0)	18 (23.7)	0.281
Married, *n* (%)	122 (76.3)	66 (78.6)	56 (73.7)	0.387
Extra-intestinal manifestations, *n* (%)	24 (15.0)	12 (14.3)	12 (15.8)	0.817
Sigmoidectomy, *n* (%)	4 (2.5)	4 (4.8)	0	0.152
Right hemicolectomy, *n* (%)	27 (16.9)	13 (15.5)	14 (18.4)	0.646
Left hemicolectomy, *n* (%)	3 (1.9)	2 (2.4)	1 (1.3)	0.612
Total abdominal or segmental colectomy, *n* (%)	7 (4.4)	2 (2.4)	5 (6.6)	0.373
Ileocolectomy, *n* (%)	61 (38.1)	39 (46.4)	22 (28.9)	0.020
Small bowel resection, *n* (%)	58 (36.3)	24 (28.57)	34 (41.6)	0.040
Medication history of CD, *n* (%)				
5-ASA	98 (61.3)	51 (60.7)	47 (61.8)	0.966
Corticosteroids	55 (34.4)	27 (32.1)	28 (36.8)	0.573
Thiopurine	75 (46.9)	37 (44.0)	38 (50.0)	0.500
Infliximab	56 (35.0)	29 (34.5)	27 (35.5)	0.943
Others	19 (11.9)	10 (11.9)	9 (11.8)	0.966
Current treatment, *n* (%)				
5-ASA	25 (15.6)	11 (13.1)	14 (18.4)	0.373
Corticosteroids	15 (9.4)	5 (6.0)	10 (13.2)	0.125
Thiopurine	64 (40.0)	32 (38.1)	32 (42.1)	0.511
Infliximab	71 (44.4)	41 (48.8)	30 (39.5)	0.209
Nutrition therapy	79 (49.4)	35 (41.7)	44 (57.9)	0.033
Micronutrient	39 (24.4)	21 (25.0)	18 (23.7)	0.810
Vitamin	53 (33.1)	26 (31.0)	27 (35.5)	0.579
Others	16 (10.0)	9 (10.7)	7 (9.2)	0.731

CD, Crohn’s disease; 5-ASA, 5-aminosalicylicacid.

### Prevalence of malnutrition in postoperative CD patients

**Figure 2:**
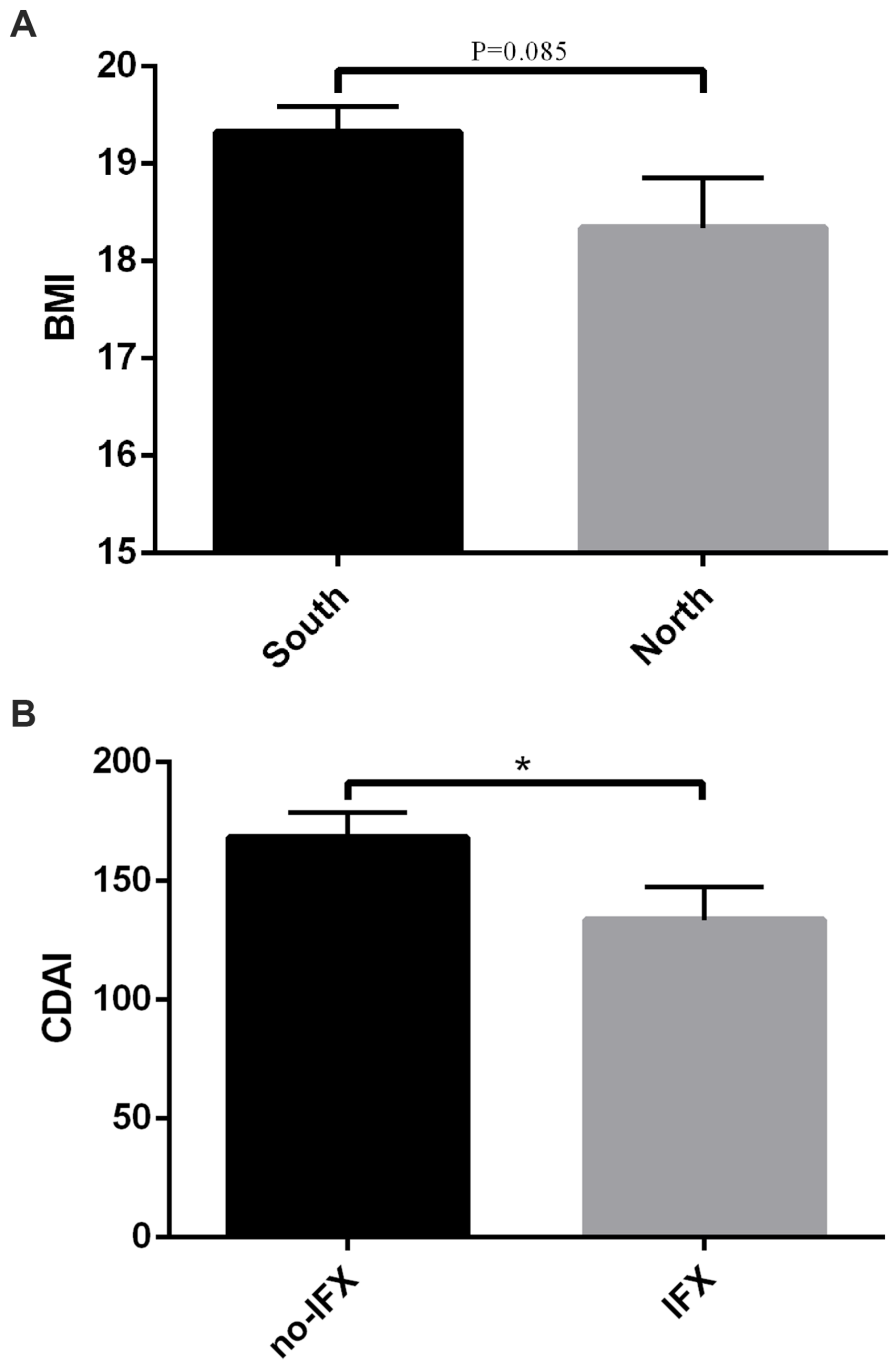
**(A)** The BMI of CD patients from Southern region and North region. **(B)** CDAI score of CD patients treated with infliximab and without infliximab. BMI, body mass index; CDAI, Crohn’s disease activity index; CD, Crohn’s disease. *P < 0.05.

The prevalence of malnutrition among CD patients and a history of surgical resection was 47.5% (76/160). According to NRS-2002, 56.3% (90/160) CD patients were at risk of malnutrition postoperatively. Among all enrolled patients, 46.3% (74/160) were underweight, 49.4% (79/160) had normal weight, 4.4% (7/160) were overweight, and none were obese, as classified by BMI. Among the malnourished patients, 46 (60.5%) were male and 30 (39.5%) were female. The most common disease location and surgical procedure in this subgroup were ileocolonic (65.8%) and small bowel resection (41.6%), respectively. Prior to surgery, most malnourished patients had a history of treatment with 5-ASA (61.8%) and thiopurine (50.0%), as detailed in Table 1. Postoperatively, the majority received thiopurine (42.1%), infliximab (39.5%), and nutrition therapy (57.9%). Regional disparities in malnutrition prevalence were evident, with a lower rate in Southern China compared with Northern China (43.3% vs. 63.6%, *p* = 0.037). Furthermore, patients from the Southern region exhibited higher ALB levels (39.9 ± 0.6 vs. 35.7 ± 1.1, *p* = 0.001) and a trend toward higher BMI (19.3 ± 0.3 vs. 18.3 ± 0.5, *p* = 0.085) (Figure 2A).

### Assessment of biochemical parameters in malnourished CD patients during follow-up after surgery

Malnourished CD patients exhibited significantly lower levels of ALB (40.5 ± 0.66 vs. 37.4 ± 0.78, *p* = 0.003), PAB (23.5 ± 1.1 vs. 18.2 ± 1.1, *p* = 0.001), and BMI (21.1 ± 0.27 vs. 16.9 ± 0.16, *p* < 0.001). The CDAI was higher in malnourished patients than in well-nourished controls (111.5 ± 9.3 vs. 194.9 ± 12.0, *p* < 0.001), and ESR level was also elevated in those with malnutrition (18.45 ± 2.49 vs. 27.16 ± 3.58, *p* = 0.043). Hemoglobin levels were lower in malnourished CD patients (124.0 ± 2.35 vs. 113.0 ± 2.71, *p* = 0.002), underscoring poorer nutritional status and more pronounced inflammation (Table 2). Although the incidence of malnutrition did not differ significantly between patients treated with and without infliximab (42.3% vs. 51.7%, *p* = 0.235), those receiving infliximab had higher ALB levels (40.4 ± 0.7 vs. 37.9 ± 0.7, *p* = 0.016). Furthermore, CDAI scores were lower in infliximab-treated patients compared to those not receiving it (168.2 ± 10.3 vs. 133.5 ± 13.8, *p* = 0.042) (Figure 2B).

**Table 2:** Comparison of biochemical parameters between patients in the normal and malnourished CD patients.

Variable	Normal (84)	Malnourished (76)	*p*-value
C-reactive protein, mg/L	16.64 ± 4.31	19.73 ± 4.13	0.607
Erythrocyte sedimentation rate, mm/h	18.45 ± 2.49	27.16 ± 3.58	0.043
Red blood cell, ×10^12^/L	4.86 ± 0.34	9.74 ± 5.55	0.364
Hematocrit, %	42.87 ± 4.99	32.80 ± 1.20	0.060
Hemoglobin, g/L	124.0 ± 2.35	113.0 ± 2.71	0.002
White blood cell, ×10^9^/L	6.43 ± 0.53	5.45 ± 0.23	0.102
Neutrophil, ×10^9^/L	18.30 ± 2.85	11.68 ± 2.46	0.083
Lymphocyte, ×10^9^/L	2.29 ± 0.14	2.46 ± 0.19	0.452
Platelet, ×10^9^/L	164.7 ± 9.4	154.3 ± 10.3	0.458
Albumin, g/L	40.5 ± 0.66	37.4 ± 0.78	0.003
Pre-albumin, mg/L	23.5 ± 1.1	18.2 ± 1.1	0.001
Ca, mmol/L	2.28 ± 0.02	2.20 ± 0.03	0.019
BMI, kg/m^2^	21.1 ± 0.27	16.9 ± 0.16	<0.001
FFMI, kg/m^2^	17.1 ± 0.89	13.8 ± 0.60	0.009
IBDQ, score	156.5 ± 5.8	146.8 ± 4.9	0.203
CDAI, score	111.5 ± 9.3	194.9 ± 12.0	<0.001
NRS-2002 ≥ 3, *n* (%)	16 (19.0)	74 (97.4)	<0.001

CD, Crohn’s disease; BMI, body mass index; FFMI, fat-free mass index; IBDQ, inflammatory bowel disease questionnaire; CDAI, Crohn’s disease activity index; NRS, nutritional risk screening.

### Prevalence of anemia in CD patients after surgery and evaluating basic characteristics of them

According to the WHO definition of anemia, 97 (60.6%) patients experienced an episode of anemia following surgical resection during follow-up, with a prevalence of 61.9% among men. Among anemic patients, 79.4% had mild anemia, and 20.6% had moderate anemia; no cases of severe anemia were reported. CD patients classified as anemic exhibited a higher prevalence of perianal disease (15.9% vs. 35.1%, *p* = 0.008). Ileocolectomy was more frequent in anemic patients compared to controls (47.6% vs. 32.0%, *p* = 0.046). Additionally, CD patients with anemia were more likely to have a history of thiopurine use (36.5% vs. 39.2%, *p* = 0.034). Significant differences in current treatments were observed between anemic and non-anemic patients, including corticosteroids use (3.2% vs. 13.4%, *p* = 0.030) and vitamin supplementation (23.8% vs. 39.2%, *p* = 0.044) (Table 3).

**Table 3:** Baseline characteristics of the patients with and without anemia.

Variable	Total (160)	Normal (63)	Anemia (97)	*p*-value
Male, *n* (%)	102 (63.8)	42 (66.7)	60 (61.9)	0.536
Mean age, years	37.6 ± 1.0	38.0 ± 1.5	37.1 ± 1.3	0.656
Mean duration of CD, month	80.6 ± 5.1	78.4 ± 7.4	83.1 ± 6.9	0.648
Smokers, *n* (%)	31 (19.4)	14 (22.2)	17 (17.5)	0.463
Age (≤16), *n* (%)	2 (1.3)	0	2 (2.1)	0.520
Age (17–40), *n* (%)	99 (61.9)	41 (65.1)	58 (59.8)	0.501
Age (>40), *n* (%)	59 (36.9)	22 (34.9)	37 (38.1)	0.680
L1 (ileal), *n* (%)	46 (28.8)	18 (28.6)	28 (28.9)	0.968
L2 (colonic), *n* (%)	18 (11.3)	8 (12.7)	10 (10.3)	0.640
L3 (ilecolonic), *n* (%)	90 (56.3)	33 (52.4)	57 (58.8)	0.427
L4 (upper gastrointestinal), *n* (%)	15 (9.4)	7 (11.1)	8 (8.2)	0.544
B1 (failure of medical therapy), *n* (%)	35 (21.9)	14 (22.2)	21 (21.6)	0.932
B2 (structuring), *n* (%)	59 (36.9)	25 (39.7)	34 (35.1)	0.553
B3 (penetrating), *n* (%)	53 (33.1)	18 (28.6)	35 (36.1)	0.324
B2 + B3, *n* (%)	13 (8.1)	6 (9.5)	7 (7.2)	0.602
Perianal disease, *n* (%)	44 (27.5)	10 (15.9)	34 (35.1)	0.008
Married, *n* (%)	122 (76.3)	46 (73.0)	76 (78.4)	0.438
Extra-intestinal manifestations, *n* (%)	24 (15.0)	9 (14.3)	15 (15.5)	0.838
Sigmoidectomy, *n* (%)	4 (2.5)	2 (3.2)	2 (2.1)	0.660
Right hemicolectomy, *n* (%)	27 (16.9)	7 (11.1)	20 (20.6)	0.117
Left hemicolectomy, *n* (%)	3 (1.9)	0	3 (3.1)	0.416
Total abdominal or segmental colectomy, *n* (%)	7 (4.4)	3 (4.8)	4 (4.1)	0.847
Ileocolectomy, *n* (%)	61 (38.1)	30 (47.6)	31 (32.0)	0.046
Small bowel resection, *n* (%)	58 (36.3)	21 (33.3)	37 (38.1)	0.536
Medication history of CD, *n* (%)				
5-ASA	98 (61.3)	37 (58.7)	61 (62.9)	0.598
Corticosteroids	55 (34.4)	17 (27.0)	38 (39.2)	0.113
Thiopurine	75 (46.9)	23 (36.5)	52 (39.2)	0.034
Infliximab	56 (35.0)	25 (39.7)	31 (32.0)	0.317
Others	19 (11.9)	8 (12.7)	11 (11.3)	0.795
Current treatment, *n* (%)				
5-ASA	25 (15.6)	10 (15.9)	15 (15.5)	0.944
Corticosteroids	15 (9.4)	2 (3.2)	13 (13.4)	0.030
Thiopurine	64 (40.0)	23 (36.5)	32 (33.0)	0.647
Infliximab	71 (44.4)	31 (49.2)	40 (41.2)	0.322
Nutrition therapy	79 (49.4)	26 (41.3)	53 (54.6)	0.098
Micronutrient	39 (24.4)	13 (20.6)	26 (26.8)	0.375
Vitamin	53 (33.1)	15 (23.8)	38 (39.2)	0.044
Others	16 (10.0)	7 (11.1)	9 (9.3)	0.706

CD, Crohn’s disease; 5-ASA, 5-aminosalicylicacid.

### Assessment of biochemical parameters in anemia CD patients during follow-up after surgery

Among CD patients with anemia, laboratory data were compared to evaluate differences in nutritional and inflammatory status. Anemic CD patients exhibited significantly higher levels of CRP (5.23 ± 0.80 vs. 26.16 ± 4.66, *p* < 0.001), ESR (10.03 ± 1.24 vs. 30.48 ± 3.16, *p* < 0.001), lymphocytes (2.04 ± 0.09 vs. 2.60 ± 0.18, *p* = 0.016), and CDAI (98.0 ± 9.6 vs. 189.1 ± 10.6, *p* < 0.001) compared to controls. Furthermore, anemic patients were more likely to be malnourished, with significantly lower levels of ALB (43.8 ± 0.57 vs. 36.7 ± 0.62, *p* < 0.001), PAB (25.8 ± 0.9 vs. 18.0 ± 1.0, *p* < 0.001), Ca (2.33 ± 0.02 vs. 2.19 ± 0.03, *p* < 0.001), and BMI (19.8 ± 0.39 vs. 18.7 ± 0.28, *p* = 0.014). Anemic patients also reported a poorer quality of life (160.4 ± 5.9 vs. 145.2 ± 4.7, *p* = 0.048) (Table 4).

**Table 4:** Comparison of biochemical parameters between patients in the normal and anemia CD patients.

Variable	Normal (63)	Anemia (97)	*p*-value
C-reactive protein, mg/L	5.23 ± 0.80	26.16 ± 4.66	<0.001
Erythrocyte sedimentation rate, mm/h	10.03 ± 1.24	30.48 ± 3.16	<0.001
Red blood cell, ×10^12^/L	4.88 ± 0.06	8.73 ± 4.43	0.483
Hematocrit, %	48.17 ± 6.48	31.38 ± 0.81	0.002
Hemoglobin, g/L	141.3 ± 1.51	104.1 ± 1.59	<0.001
White blood cell, ×10^9^/L	6.45 ± 0.62	5.65 ± 0.28	0.192
Neutrophil, ×10^9^/L	18.67 ± 3.32	12.79 ± 2.26	0.132
Lymphocyte, ×10^9^/L	2.04 ± 0.09	2.60 ± 0.18	0.016
Platelet, ×10^9^/L	173.6 ± 9.8	150.5 ± 9.5	0.104
Albumin, g/L	43.0 ± 0.67	36.4 ± 0.61	<0.001
Pre-albumin, mg/L	25.8 ± 0.9	18.0 ± 1.0	<0.001
Ca, mmol/L	2.33 ± 0.02	2.19 ± 0.03	<0.001
BMI, kg/m^2^	19.8 ± 0.39	18.7 ± 0.28	0.014
IBDQ, score	160.4 ± 5.9	145.2 ± 4.7	0.048
CDAI, score	98.0 ± 9.6	189.1 ± 10.6	<0.001
NRS-2002 ≥ 3, *n* (%)	24 (38.1)	66 (68.0)	<0.001

CD, Crohn’s disease; BMI, body mass index; FFMI, fat-free mass index; IBDQ, inflammatory bowel disease questionnaire; CDAI, Crohn’s disease activity index; NRS, nutritional risk screening.

## Discussion

Numerous studies have investigated the nutritional status of Crohn’s disease (CD) patients, identifying malnutrition as a well-recognized complication of the condition. However, reported prevalence rates of malnutrition vary widely, ranging from 20 to 85% (15). Prior research has explored malnutrition in both pediatric and elderly populations (16, 17). In this study, we evaluated the nutritional status of CD patients following surgical resection. To our knowledge, this is the first study to assess the nutritional status of these patients during post-surgical follow-up. Given the strong association between anemia and nutritional status (18), we also investigated the prevalence of anemia in CD patients after surgery.

Previous studies have shown that almost 70–80% of CD patients will undergo CD-related surgery during their lifetime (19). While emerging research has focused on the nutritional status of CD patients, few studies have specifically investigated nutritional outcomes in post-surgical CD patients. Our study provides initial evidence demonstrating that the rate of malnutrition in this cohort is consistent with prior reports (20). Notably, same studies reported even lower malnutrition rates than ours, which we attribute to differences in patient populations. Our cohort exclusively included CD patients who had undergone surgical resection (5). This highlights the importance of prioritizing nutritional assessment in post-surgical CD patients. We further found that ileocolectomy and small bowel resection were associated with an increased risk of malnutrition. Animal models indicate that preserving the ileocecal valve promotes weight gain (21). Thus, ileocolectomy in CD patients may contribute to weight loss. Small bowel resection, on the other hand, impairs the digestive and absorptive functions of the small intestine, further exacerbating malnutrition. Our study also revealed a correlation between malnutrition and CDAI scores. Gong et al. found that CD patients in remission had better nutrition status, whereas BMI was lower in those with active disease (22). These findings align with our data on post-surgical nutritional status, reinforcing the consistency of nutritional trends in CD patients across different cohorts.

The current dogma of CD pathogenesis includes environment factors, genes and immune system (23). Among these, current evidence suggests that environmental exposures play a crucial role in CD development, particularly dietary factors in disease epidemiology (24). A Western dietary pattern is reported to be associated with an increased risk of CD (25). Diet may influence gut health directly by affecting gut barrier function and homeostasis, or indirectly through its impact on the gut microbiome (24). Many CD patients perceive certain foods as triggers for disease flares, leading them to adopt restrictive eating behaviors that can result in malnutrition (6). In our study, we compared the prevalence of malnutrition between Southern and Northern Chinese region in post-surgical CD patients. It is well established that dietary patterns vary significantly between different regions of China, particularly between the South and North. Our results revealed that the prevalence of malnutrition in North was found to be higher based on measurements of ALB and BMI. Southern China’s higher levels of urbanization and economic development may contribute to better access to quality healthcare, which in turn could improve the nutritional status of CD patients. For example, northern and southern regions differ in terms of income levels, industrial structure, supply chains, and nutritional literacy. Southern urban populations tend to prioritize food quality over quantity, whereas northern rural areas often have limited access to fresh produce.

Although various medical therapies including immunosuppressive and biologic agents have emerged in recent years, surgical resection remains necessary for CD patients with medical therapy failure (26). Approximately 30% of CD patients require surgical resection within 5 years of diagnosis, and 70–80% will undergo CD-related surgery during their lifetime (19). However, surgery is not curative for CD, and thus post-surgical medical therapy remains essential. Tumor necrosis factor (TNF)-α monoclonal antibodies, such as infliximab, are widely used to induce and maintain remission in moderate to severe CD (27). To our knowledge, data on the impact of these therapies on post-surgical nutritional parameters are limited. In our study, we found that CD patients treated with infliximab postoperatively had lower CDAI score, higher BMI, and higher ALB level. These findings suggest that infliximab better controls disease activity. When patients achieve remission, they can eat without pain, nausea, or vomiting, which directly improves nutritional status. Our results are consistent with previous studies demonstrating that infliximab reduces inflammation, alleviates disease symptoms, and promotes mucosal healing, thereby enhancing nutritional status and body weight in post-surgical CD patients (28). What’s more, ALB acts as both a marker of nutritional status and systemic inflammation, we also analyzed ALB level between patients with CDAI >150 and patients with CDAI≤150, we found the ALB level was higher in patients with CDAI≤150 (41.5 ± 5.6 g/L vs. 38.0 ± 6.5 g/L, *p* = 0.007). Thus, disease activity is associated with ALB level, which indicated that other nutrition markers are needed in the future.

In previous studies exploring the epidemiology of anemia in CD patients, limited data have focused on the prevalence of anemia in surgical patients during follow-up. In the current study, we found a higher prevalence of anemia than reported in prior research (7). According to other studies, several factors may contribute to the high incidence of anemia in CD patients: (1) vitamin B_12_ and folate deficiency resulting from severe small intestine inflammation or following ileocolectomy; (2) hepcidin upregulation due to increased cytokine production, which inhibits intestinal iron absorption; (3) medications such as thiopurines and methotrexate, which suppress erythropoiesis; and (4) chronic blood loss from uncontrolled disease activity (8). Our findings were consistent with previous work, demonstrating a significantly association between anemia and ileocolectomy. Other studies suggested that the most common surgery types associated with anemia were ileocolectomy and small bowel resection (8, 21). Additionally, post-surgical CD patients with anemia typically exhibited higher CDAI scores and more severe inflammation, suggesting that disease activity possible plays a key role in influencing anemia risk.

However, several limitations of this study should be acknowledged. First, the sample size of CD patients with a surgical history was relatively small, as most hospitalized CD patients receive pharmacological or conservative treatment. A multi-center design would be warranted in future studies to improve the generalizability of our findings. Second, nutritional status was not evaluated using body composition analysis, which could provide more comprehensive insights into muscle mass, fat distribution, and overall nutritional adequacy. Maybe the evaluation of body composition may better correlate with clinical outcomes. Third, disease activity was assessed solely via clinical criteria, whereas endoscopic evaluation would offer more objective and sensitive data on mucosal inflammation, which is critical for guiding treatment decisions. Fourth, detailed dietary compositions data were lacking. While future studies will investigate the association between diet and nutritional factors in CD, dietary analysis in China presents unique challenges due to the vast diversity of regional dietary habits. Finally, the assessment of anemia is incomplete, such as serum iron and folate. Iron deficiency or chronic disease also need to be identified as causes of anemia in future.

In conclusion, malnutrition is highly prevalent among CD patients following surgery. Disease activity consistently impacts nutritional status, highlighting the need for surgeons to emphasize the significance of perioperative management, particularly postoperative medical therapy. Additionally, we observed a high prevalence of anemia among CD patients after surgery in China. To address this gap, anemia management following surgery should be more rigorously implemented in accordance with established clinical guidelines.

## Data Availability

The raw data supporting the conclusions of this article will be made available by the authors, without undue reservation.
